# Viral Manipulation of the Host Epigenome as a Driver of Virus-Induced Oncogenesis

**DOI:** 10.3390/microorganisms9061179

**Published:** 2021-05-30

**Authors:** Shimaa Hassan AbdelAziz Soliman, Arturo Orlacchio, Fabio Verginelli

**Affiliations:** 1 Simpson Querrey Institute for Epigenetics, Department of Biochemistry and Molecular Genetics, Northwestern University Feinberg School of Medicine, Chicago, IL 60611, USA; 2 Department of Cancer Biology and Genetics, College of Medicine, The Ohio State University, Columbus, OH 43210, USA; arturo.orlacchio@osumc.edu; 3 Arthur G. James Comprehensive Cancer Center, The Ohio State University, Columbus, OH 43210, USA; 4 Department of Pharmacy and Center for Advanced Studies (CAST), G. d’Annunzio University of Chieti-Pescara, 66100 Chieti, Italy

**Keywords:** oncogenic viruses, epigenetic, HPV, HBV, HCV, MCPyV, KSHV, HTLV-1, EBV, HCMV

## Abstract

Tumorigenesis due to viral infection accounts for a high fraction of the total global cancer burden (15–20%) of all human cancers. A comprehensive understanding of the mechanisms by which viral infection leads to tumor development is extremely important. One of the main mechanisms by which viruses induce host cell proliferation programs is through controlling the host’s epigenetic machinery. In this review, we dissect the epigenetic pathways through which oncogenic viruses can integrate their genome into host cell chromosomes and lead to tumor progression. In addition, we highlight the potential use of drugs based on histone modifiers in reducing the global impact of cancer development due to viral infection.

## 1. Introduction

Viral infections account for an estimated 15–20% of global human cancer cases [1,2]. However, it took several years to undoubtedly acknowledge the contagious nature of tumors developed through viral infection. In 1909, Francis Peyton Rous performed his famous experiment on a sarcomatous breast tumor from a Plymouth chicken. He injected a cell-free tumor extract in a normal chicken of the same breed. Two years later, the chicken developed sarcoma [3]. This experiment was not totally accepted, due to the fact that avian tumors were considered different from human tumors. However, 50 years later, Rous was recognized for his work on tumor transmission through viral infection and in 1966 he was awarded the Nobel prize for Physiology and Medicine [4].

The first direct evidence that human tumors can be induced by and transmitted through viruses emerged in 1964. Electron microscopy studies performed by Epstein and Barr led to the discovery of viral particles in Burkitt’s lymphoma [5,6]. Later on, Werner and Gertrude Henle further corroborated the connection between the Epstein–Barr Virus (EBV) and Burkitt’s lymphoma when they discovered that EBV can directly immortalize B cells after infection [7]. Successively, Harald zur Hausen, who was awarded the Nobel prize in 2008, demonstrated that human papillomavirus (HPV) cause cervical cancer [8]. Furthermore, several studies showed different strains of oncogenic viruses that could result in tumors in specific tissue or have a broad range of tissues to infect and transform their cells [9].

Oncogenic viruses range from RNA viruses such as human T-lymphotropic virus-I (HTLV-I) and Hepatitis C virus (HCV) to a variety of DNA viruses, such as Epstein–Barr virus (EBV), Hepatitis B virus (HBV), human papillomaviruses (HPV), Kaposi’s sarcoma herpesvirus (KSHV), and Merkel cell polyomavirus (MCPyV) [10].

As of now, the exact mechanisms by which viruses lead to tumor development are not fully understood. Several studies demonstrated that viral infection is not sufficient for cancer development by itself [11,12] but that they contribute to oncogenesis through oncogenic viral protein production, chronic inflammation, and deregulation of host cell homeostasis [2,13,14,15,16]. Epigenetic alterations provide a common mechanism of virus-induced transformation. Indeed, viral encoded proteins as well as viral non-coding RNAs, such as long and small non-coding RNAs including miRNAs and circular RNAs, induce host epigenetic modifications that alter gene expression without affecting the genomic sequence of the DNA [17,18,19,20,21,22,23]. The first evidence that epigenetic alterations could lead to cancer development emerged from the studies of Feinberg and Vogelstein, who demonstrated that colorectal cancer harbors altered gene methylation patterns compared to normal tissues [24].

Reversibility and heritability are outstanding features of epigenetic modifications [25]. Therefore, several therapeutic strategies target proteins involved in epigenetic modifications. These epigenetic regulations include DNA methylation, specifically at promoter regions and CpG islands, histone post-translational modifications (PTMs), and chromatin 3D structure including promoter-enhancer looping [26,27,28,29].

Several studies show that all seven known human oncogenic viruses seduce the host epigenetic machineries by expression of viral proteins and ncRNAS to generate optimal gene expression programs that favor viral integration, latency, replication, and, in some cases, tumorigenesis [30,31,32,33,34]. Notably, viral ncRNAs have recently been studied by many researchers due to their high epigenetic or post-transcriptional regulatory effect on transcriptional induction. It is known that ncRNAs, in particular viral miRNAs and circRNAs, play a fundamental role in switching from lytic to latent phases, virus persistence and cell survival. Furthermore, viral circRNAs are expressed in infected cells but can be secreted into peripheral blood or transferred by extracellular vesicles to other cells to favor the spread of infection, with the advantage of being less recognizable by the host’s immune system [35]. In spite of the multitude of ncRNAs encoded by viruses, only a small fraction have been functionally and structurally characterized. Further understanding of underlying mechanisms related to viral ncRNAs expression could allow their use as potential biomarkers of disease and therapeutic targets [23,36].

Here, we focus mainly on the description of the epigenetic mechanisms controlled by the most relevant viral oncoproteins encoded by the identified human onco-viruses and their genome-wide effects on gene expression patterns. Furthermore, this review considers the potential oncogenic role of human cytomegalovirus (HCMV), not yet included in the group of oncogenic viruses although the available data may support this hypothesis. In addition, we discuss the current therapeutic strategies which target epigenetic modifications/enzymes and a future prospective toward the reversion of oncogenic signatures implemented by oncogenic viruses.

## 2. Hepatitis C Virus (HCV)

Hepatocellular carcinoma (HCC) is the second leading cause of cancer-related death worldwide. Unfortunately HCV infection is growing worldwide, and the infection rate tripled in the USA from 2010 to 2016 [37,38]. As a heterogeneous disease, there are several factors that are involved in the development of HCC. Nonetheless, the main factor is HCV infection which occurs in 80% of HCC cases [39,40].

HCV is an enveloped RNA virus with little potential to integrate its genome into the host cell genome [41,42,43]. Its positive-strand RNA genome encodes a single polyprotein cleaved by host and viral proteases [44,45]. The non-structural proteins play an important role in viral replication, budding, and assembly, while the structural ones such as core protein C and envelope proteins E1 and E2 form the viral particles that encapsulate the viral genome [46]. HCV infection is associated with long term inflammation and cirrhosis. However, several studies show that inflammation by itself is not sufficient for HCC development [47,48,49].

Several therapeutic strategies have been developed to eliminate HCV infection. Among these, direct-acting antivirals (DAAs) represent a major breakthrough in viral eradication [50,51,52]. However, these therapies do not eliminate the virus-induced HCC risk, especially in patients with liver cirrhosis [53,54]. Several lines of evidence demonstrate that specific epigenetic signatures induced by HCV infection result in a “lasting” epigenetic memory, which persists after viral eradication. This permanent “scarring” suggests a novel mechanism for the pathogenesis of HCV even after its eradication with DAAs [55,56,57,58].

HCV infection can influence the epigenetic status of the host DNA through a combination of direct and indirect factors. Genome-wide analysis using chromatin immunoprecipitation followed by deep sequencing (ChIP-Seq) and RNA sequencing (RNA-Seq) of chronically HCV-infected liver tissues showed global changes in histone H3 lysine 27 acetylation (H3K27ac) levels, which are markers of active enhancers, which correlated with elevated expression of cancer-related genes [59]. Interestingly, H3K27ac profiles in HCV-cured fibrotic patient livers versus non-fibrotic HCV-cured chimeric mice yielded an HCV-specific persistent epigenetic and transcriptomic “footprint” of 65 cancer genes in fibrotic tissues. Recently, Hamdane et al. showed that epigenetic changes in H3K27ac levels are induced through direct interaction between HCV and hepatocytes and indirectly through liver fibrosis [59]. Importantly, several studies show that these changes persist after sustained viral response (SVR) to either DAAs or interferon-based therapies [60,61,62].

Altogether, these studies demonstrate that altered H3K27ac histone modification induced by HCV infection is a causal factor for HCC risk even after DAA cure.

Similarly, in vitro infection with HCV resulted in changes in the activation mark histone H3 lysine 9 acetylation (H3K9ac). Remarkably, in vitro treatments with drugs such as C646, a specific inhibitor of H3K9ac, reverted the HCV-induced epigenetic alterations, thus preventing oncogenesis [33]. These studies suggest a “hit and run” strategy that could partially explain why some HCV cured patients develop HCC following viral eradication.

HCV does not only alter the genome-wide acetylation levels but also plays a fundamental role in regulating the host DNA methyltransferases. The HCV core protein increases the levels of the maintenance methyltransferase DNMT-1 and the de novo methyltransferase DNMT3B, causing epigenetic silencing of host tumor suppressor genes through methylation of cytosine-phospho-guanine (CpG) dinucleotides in regulatory elements. Examples of tumor suppressor genes silenced by HCV infection include the secreted frizzled-related protein (SFRP) gene whose product deregulates the Wnt/ß-catenin signaling pathway involved in HCC development. In addition, DNA hypermethylation was observed in HCC tumors at specific genes such as Ras Association Domain Family Member 1 (RASSF1A), Glutathione S-transferases (GSTP1), Neuronal acetylcholine receptor subunit alpha-3 (CHRNA3), and Docking protein 1 (DOK1) compared to normal or cirrhotic tissues [63].

Additionally, Wijetunga et al. demonstrated that HCV-infected liver tissues are hyper-methylated at active enhancer regions enriched for the binding of transcription factors Forkhead Box Protein A1 (FOXA1), Forkhead Box Protein A1 (FOXA2), and Hepatocyte Nuclear Factors 4 alpha (HNF4A) and that correlated with reduced expression of genes involved in liver cancer as stem cell phenotype development [64].

A study performed by Perez et al. showed that in Huh7.5 cells, HCV infection greatly affects the levels of H3K4me3 over 1200 genomic regions, and H3K9me3 levels over 9000 genomic regions.

HCV infection results in proteasomal degradation of the E3 ubiquitin-protein ligase RING2 protein (encoded by RNF2), a component of the Polycomb Repressor Complex 1 (PRC1).Therefore, a decrease in the monoubiquitination of K119 H2A (K119H2Aub)classically targets the homeobox (HOX) genes, whose expression is deregulated in tumors [65]. As a result, more than half of HOX genes levels are upregulated. Interestingly, degradation of RNF2 is also viral core protein dependent. However, is still unknown if HOX genes upregulation in HCV infected cells is sufficient to drive HCC.

A potential explanation is that deubiquitination of H2A results in the recruitment of the Facilitates Chromatin Translocation (FACT) complex, therefore, promoting transcriptional elongation of the RNA polymerase II (Pol II) through the HOX gene bodies. Indeed, several diseases show genome wide defects in Pol II elongation rates over a wide range of genes due to defects on histone post-translational modifications.

Therefore, the study of the Pol II elongation machinery in infected cells could potentially lead to the discovery of new targets in the treatment of virus induced tumors.

## 3. Human T-Cell Lymphotropic Virus 1 (HTLV-1)

Human T-lymphotropic virus type 1 (HTLV-1) is a single-stranded RNA virus belonging to the Retroviridae family and was the first human retrovirus to be discovered [66].

HTLV-1 is responsible for the oncogenic transformation of CD4^+^ T-cells that cause adult T-cell leukemia/lymphoma (ATL) in about 3–5% of infected individuals [67,68,69]. The main route of viral transmission is breastfeeding, however infection through sexual intercourse or exposure to infected blood is also possible.

Similar to other retroviruses, after viral entry the viral RNA genome is converted into a double-stranded DNA molecule through reverse transcription and moves to the nucleus due to its association with different viral proteins. Successively, the pro-viral DNA integrates into the host genome. Sequences of the structural proteins gag, pol, and env are present in the protein-encoding region of the HTLV-1 pro-viral genome.

Two long terminal repeat sequences (LTRs) flank the protein coding region, while between the *env* gene and the 3′-LTR a region named pX is present and encodes for the viral regulatory factors Tax, Rex, p12, p13, p30 and p21 and HTLV-1 basic leucine zipper factor (HBZ). Furthermore, the 5′ LTR contains the main promoter that drives viral gene transcription [70,71]. Among these regulatory viral factors, Tax and HBZ are thought to play the main role in tumor development [70,72].

Tax can induce the degradation of the α and β subunits of the NF-κB inhibitor IκB. At the same time, Tax can bind to IKK-γ, the non-catalytic subunit of the IκB kinase (IKK), leading to activation of the catalytic subunits IKK-α and IKK-β. This translates into the negation of the inhibitory action of IκB [72]. Moreover, there is evidence that Tax can recruit IKK-α to the NF-κB subunit p100, thus triggering its phosphorylation-dependent ubiquitylation and processing, converting it to the p52 subunit of NF-κB [73].

Recent work from Ameur and colleagues has shown that the NF-κB subunit p65 is recruited to intragenic regions to regulate alternative splicing upon Tax-induced NF-κB activation. Specifically, p65 directly regulates splicing by binding to gene sequences in the proximity of GC-rich exons and recruits the splicing factor DEAD-Box Helicase 17 (DDX17). Even though the effect on splicing mediated by p65 is not dependent on Tax, this viral factor dramatically shifts the balance in its favor. Interestingly, Tax-regulated alternatively spliced transcripts were found to be enriched in different functional pathways when compared to those enriched by Tax through transcriptional effects. This suggests that splicing reprogramming may represent a separate regulative mechanism employed by HTLV-1 in order to alter the host transcriptome [74].

Tax can also inhibit the expression of the tumor suppressor BRCA1 [75]. Under normal conditions, this gene is expressed upon the binding of estrogen (E2) to its receptor (ERa) and the formation of a complex with CBP/P300, which then binds to the BRCA1 promoter [76]. Tax binds to the ERa-CBP/P300 complex, thus preventing its binding to the BRCA1 promoter. It has been suggested that, given the importance of BRCA1 inactivation in the development of breast cancer, HTLV-1 could be involved in this process as well [77].

A recent retrospective study, however, compared breast cancer patients with and without HTLV-1 infection and didn’t find meaningful differences in disease-free survival, overall survival rates or any other clinicopathological factor [78].

Other genes that can bind Tax are histone acetyltransferases and protein arginine methyltransferase 4 (PRMT4, also known as CARM1). It has been demonstrated that Tax can induce transcription by inducing histone acetylation and acetylation-dependent dislodgment of the entire octamer through a process involving the histone chaperone nucleosome assembly protein (NAP1) [79,80].

Tax can negatively affect the expression of SH2-homology containing protein-tyrosine phosphatase 1 (SHP-1) by recruiting HDAC1 to its promoter, thus causing the displacement of the NF-κB transcription factor [81].

Tax expression is often lost in aggressive ATL forms due to repressive hypermethylation of the viral promoter. The viral protein HBZ, however, has been found to be expressed at both early and later stages [82].

Similar to Tax, HBZ causes deregulation of a number of signaling pathways by interacting with different transcription factors [83,84,85,86,87]. Interestingly, it has been reported that HBZ is not able to form stable homodimers and therefore, in order to affect gene transcription, has to form heterodimers with other proteins [88].

HBZ can specifically inhibit the classical NF-κB pathway by reducing the DNA binding of p65 and by targeting the same protein to degradation though the PDZ-LIM domain-containing protein 2 (PDLIM2) [85]. At the same time it has been reported that Tax-induced chronic NF-κB hyper-activation can lead to cellular senescence mediated by cellular p21-/27 [89]. Therefore, the inhibitory effect of HBZ on the classical NF-κB pathway appears to help cells avoid senescence and promote proliferation.

Although Tax and HBZ are the main effector of HTLV-1 induced transformation, the pro-viral genome itself has a deep effect on host gene expression. In fact, it has been shown that the integrated HTLV-1 provirus harbors a binding site for the highly conserved zinc finger protein CCCTC-binding factor (CTCF), a key regulator of chromatin structure and function [90].

CTCF when bound to the integrated HTLV-1 provirus acts as a barrier element, dampening the effect of enhancers, while regulating HTLV-1 mRNA splicing and altering the host chromatin structure by establishing long-distance interactions within it [90]. This study was later expanded to show that the HTLV-1 provirus does form abnormal chromatin contacts with sites in cis up to 1.4 Mb from the site of integration. As a result, transcription of host loci in cis to the integrated provirus is deregulated. This effect extends as far as 300 kb from the viral integration site.

Considering that the HTLV-1 provirus integrates randomly into the host genome, and that in a host the virus infects typically between 10^4^ and 10^5^ independent T cell clones, it is easy to understand that HTLV-1 can potentially deregulate tens of thousands of host genes [91]. This highlights the importance of virus-induced alterations of the host DNA quaternary structure in the development of ATL.

## 4. Epstein–Barr Virus (EBV)

Epstein–Barr Virus (EBV), also known as Human Herpes Virus-4 (HHV-4), is a gamma-1 herpesvirus that infects about 95% of the population worldwide [92,93,94].

EBV is transmitted through the saliva, infects the oropharyngeal epithelial cells, then penetrates the mucosal barrier and goes into the blood [95]. Most infected individuals harbor asymptomatic infection in which EBV resides in naïve and memory B cells where it establishes latent infection although, in some cases, EBV could lead to lymphocyte transformation [96,97]. In 1964, Tony Epstein and Yvonne Barr detected EBV viral particles in a subpopulation of Burkitt lymphoma (BL)-derived tumor cells in vitro [5].

Infection with EBV leads to several B cell malignancies such as endemic/sporadic Burkitt lymphoma (e/sBL), Hodgkin lymphoma (HL), primary effusion lymphoma (PEL), and diffuse large B cell lymphomas (DLBCL) [98,99,100]. In addition to B cell malignancies, EBV induces epithelial tumors, including 100% of nasopharyngeal carcinoma (NPC) and 10% of gastric cancers [101,102,103,104]. Additional studies revealed that EBV has the ability to induce additional cancer types, such as T-cell lymphomas, and leiomyosarcoma [105,106,107].

The mechanisms that follow EBV infection to drive viral integration and sometimes tumorigenesis are poorly understood. EBV has a complex life cycle, showing different cellular tropisms depending on the stage of infection. At least five different stages have been described as being part of the viral life cycle: lytic infection, latency III, latency II, latency I/0, and lytic reactivation [108,109,110].

Following infection, EBV expresses lytic genes, then undergoes a latent stage in which the viral genome exists as episome with limited expression of latent proteins. During the latent stages, latent-specific proteins are responsible for ensuring the persistence of the virus but also for inducing malignant growth in immunocompromised patients.

**Figure 1:**
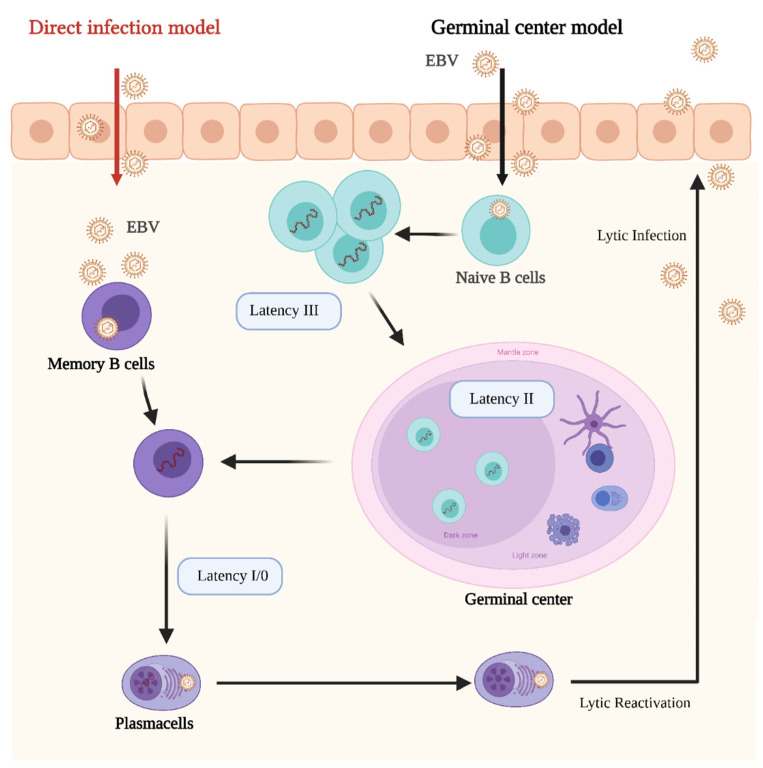
Schematic representation of the Epstein–Barr Virus (EBV) life cycle and latency stages. In the germinal center model EBV infects submucosal naive B cells and establishes the latency III program. In this stage the proliferation and expansion of the infected B cell pool is driven by the expression of all the EBV latent genes. Then, the infected cells enter in the germinal center, where they proliferate and mature. At this stage the Latency II program is established. Some infected B cells leave the germinal center as memory B cells. At this stage EBV infection is still latent (latency I/0), however, if infected memory cells differentiate in plasma cells and lytic reactivation is triggered. In the direct infection model, memory B cells are directly infected. Credits: Created with BioRender.com.

In its latent stages, EBV is able to manipulate the host’s epigenetic machinery and cellular signaling pathways. Latency in B cells can be established by direct infection of memory B cells or by infecting naïve B cells which later became memory B cells by passing through the lymph node germinal center (GC) [111] (Figure 1).

Viral reactivation mechanisms are still poorly understood. However, epigenetic alterations in the host cells are main factors for EBV lytic reactivation and tumorigenesis. Five EBV latent proteins, EBNA2, EBNALP, EBNA3A, EBNA3C, and LMP1, are essential for B cell transformation [112].

The most common epigenetic alteration detected in EBV-induced malignancies is DNA hypermethylation [113]. Global DNA methylation levels gradually increase from normal to premalignant to malignant state [114]. Such an increase in DNA methylation is mediated by the EBV latent proteins EBNA1 and EBNA2, which alter the levels of the maintenance methyltransferase DNMT1 and the de novo methyltransferases DNMT3a and 3b and suppress E-cadherin, hence increasing cell migration [115]. In addition, the latent membrane proteins LMP1 and LMP2A increase the levels of DNMT1, 3A and 3B by activating JNKs signaling pathway and the histone demethylase KDM6B, leading to demethylation of H3K27 implemented by EZH2 [116,117]. In line with these findings, depletion of EBNA1 leads to a transcriptional de-repression of silenced genes and reduction in H3K9me3. EBNA2 also tunes the methylation status of several genes that are essential for EBV-induced B cell transformation by activating the expression of the demethylase ten-eleven translocation methylcytosine dioxygenase-2 (TET2) [118,119].

EBV-driven tumorigenesis is not only involved in DNA methylation but also in histone acetylation. It was previously shown that EBNA2 interacts with the p300/CBP complex while EBNA3C interacts with the histone deacetylase (HDAC) [120]. Histone acetylation marks mainly occupy enhancer and super enhancer regions and previous studies showed that EBNA2 and EBNA3 binding sites are located outside promoter regions of host protein-coding genes. ChIP-Seq analysis of EBNA binding sites showed that these regions encompass enhancer regions. Therefore, EBNA2 and EBNA3 proteins seem to control transcription programs by targeting enhancers [121].

In addition to altering histone modification patterns, EBNA2 expression results in upregulation of the MYC proto-oncogene in B cells through binding to the enhancer and super enhancer regions around the MYC promoter [122].

Epigenetic alterations driven by EBV infection are not limited to B-cell malignancies. As an example, The Cancer Genome Atlas (TCGA) classifies gastric cancer into four major categories: EBV-associated GC (EBVaGC), genomically stable GC (GS), microsatellite instable (MSI), and GC with chromosomal instability (CIN) [123]. Gastric cancer harbors the highest levels of DNA methylation in both EBV positive tissues and MSI-high tissues [124]. Interestingly, it has recently been shown that small molecules targeting EBNA1 significatively inhibit tumor growth in EBV-positive gastric cancer xenografts but not in EBV-negative ones, thus suggesting that EBNA1 inhibitors could be explored as therapeutical approach for patients affected by EBVaGC [125].

Fiches et al. showed that EBV lytic replication leads to silencing of immune related genes (IRG) such as MT1H, HOXA10, MAL, and IRAK2 through hypermethylation [126]. In addition, diagnostic tools have been developed for detection of EBV-associated NPC. Zheng et al. reported that the degree of EBV DNA methylation and the viral DNA load can be used as diagnostic markers for NPC samples [127].

Moreover, a recent report suggests that EBV may also be involved in the development of Multiple Myeloma (MM), and probably in other types of cancer as well [128]. The authors analyzed EBV positive B-cell lines derived from MM patients and found defective viral genomes characterized by aberrant viral gene expression patterns which show gene expression signatures for bone marrow derived lymphoid stem cells.

Altogether, these studies clearly demonstrate the pivotal role of host cell epigenetic programs in supporting the lytic activation of EBV and its control by viral proteins to drive transformation.

## 5. Hepatitis B Virus (HBV)

Hepatitis B virus (HBV), a member of the *Hepadnaviridae* family, is an enveloped virus containing a partially double-stranded circular DNA genome with an approximate length of 3200 bp [129,130].

Chronic HBV infection is detected in about 292 million people worldwide and accounts for 45% of the global hepatocellular carcinoma (HCC) cases, causing about one million deaths each year [129]. Following infection, the virus genome reaches the nucleus and is converted into a more stable conformation, described as covalently closed circular DNA (cccDNA). Therefore, HBV promotes tumorigenesis mainly by integrating its genome into the host chromosomes, leading to chromosomal instability [130].

Interestingly, a recent paper showed that HBV DNA contact sites with the host genome are not randomly positioned. In fact, by employing 3C-high-throughput genome-wide translocation sequencing (3C-HTGTS), it was demonstrated that HBV DNA contacts the host genome mostly at regions enriched for H3K4me1. This histone modification is associated with actively transcribed chromatin, suggesting that this is a requirement for HBV transcription [131].

The HBV genome encodes four main genes: C (HBcAg), X (HBx), P (DNA polymerase) and S (HBsAg). The HBx protein plays a main role in controlling the host gene expression through epigenetic alterations, which are believed to be one of the main mechanisms for the development and progression of HBV-associated HCC [132]. In this context, increasing evidence shows that one of the key factors in HCC development is represented by changes in host DNA methylation patterns [133].

HBx physically interacts with DNMT3A, directing it to the regulatory promoters of genes like interleukin-4 receptor (IL4R) and metallothionein-1F (MT1F), which are silenced through DNA methylation [134]. Similarly, modification in the CpG methylation pattern of the host cell is believed to be the cause of alterations in the Rb pathway, frequently observed in HBV- associated HCC. In fact, the tumor suppressor gene p16^INK4A^ is frequently inactivated and, through analysis of the methylation status of CpG islands, it has been demonstrated that methylation levels are increased in HCC tissues [135].

It was also demonstrated that p16^INK4A^ repression through hypermethylation is due to the effect of HBx on DNMT1 and DNMT3A during the early stage of HBV- associated HCC [136]. The resulting p16^INK4A^ downregulation inhibits *pRb* and consequently upregulates *E2F1*, leading to higher DNMT1 levels, which further increase p16^INK4A^ promoter methylation [137].

Another tumor suppressor negatively affected by DMNTs- dependent methylation is Ras association domain family 1 isoform A (RASSF1A). It was reported that DNMT1 and DNMT3B can hyper-methylate CpG clusters in the RASSF1A promoter, thus leading to transcriptional silencing [138]. In line with this, a strong positive correlation between hyper-methylation of the RASSF1A promoter and tumor size was previously reported. HCC in both p16^INK4A^ and RASSF1A show increased levels of the repressive H3K9 and H3K27 methylation marks at their promoter regions [139].

In an interesting study, Yuan et al. reported that HBV infection can silence the suppressor of cytokine signaling 3 (SOCS3) gene by promoter methylation. Through ROS accumulation, HBV infection upregulates the expression of Snail Family Transcriptional Repressor 1 (SFTR1) which, in association with DNMT1 and HDAC1, mediates the epigenetic silencing of SOCS3 [140]. At the same time, ROS accumulation activates the IL-6/STAT3 pathway, which is often hyperactive in cancer [141]. SOCS3 is both a target and repressor of STAT3, creating a negative feedback. Therefore, silencing of SOCS3 results in constitutive activation of the IL-6/STAT3 pathway [142]. It is not clear if such a mechanism is mediated by HBx or by other HBV proteins.

A combined analysis of the TCGA and GEO databases identified guanine nucleotide-binding protein subunit α 14 (GNA14) as a possible tumor suppressor in HCC. The authors show that HBx can mediate the hypermethylation of the GNA14 promoter, thus reducing its expression levels. Moreover, GNA14 downregulation promoted HCC cells proliferation and metastasis in vivo and in vitro. Specifically, GMA14 down-regulation negatively affects Notch1 cleavage and promotes cell cycle progression. Moreover, GMA14 suppresses the metastatic potential of HCC by inhibiting Jumonji Domain Containing 6 (JMJD6), probably by facilitating its degradation [143].

On the other hand, several reports suggest that HBx may also have a role in the hypomethylation of specific loci of the host genome [144,145,146]. In mouse models expressing HBx in hepatocytes, HBx repressed *DNMT3A* and *DNMT3L* expression by recruiting HDAC1 to their promoters. Consequently, epigenetic modifications associated with active transcription, like H3K36me3, possibly caused abnormal cell differentiation [144].

One example of HBx-induced hypomethylation was reported in 2016 by Fan et al. These authors demonstrated that in the presence of HBx, the NF-kB subunit RelA forms a complex with EZH2, TET2 and DNMT3L causing DNA demethylation at the CpG sites of the epithelial cell adhesion gene *EpCAM* leading to its overexpression. The function of DNMT3L in this context, was not fully characterized [146]. The role of DNM3L in negatively regulating DNA methylation is also supported by a previous report [147] suggesting that DNMT3L can compete with DNMT3A and DNMT3B for the binding to PRC2, thus preventing H3K27me3.

**Figure 2:**
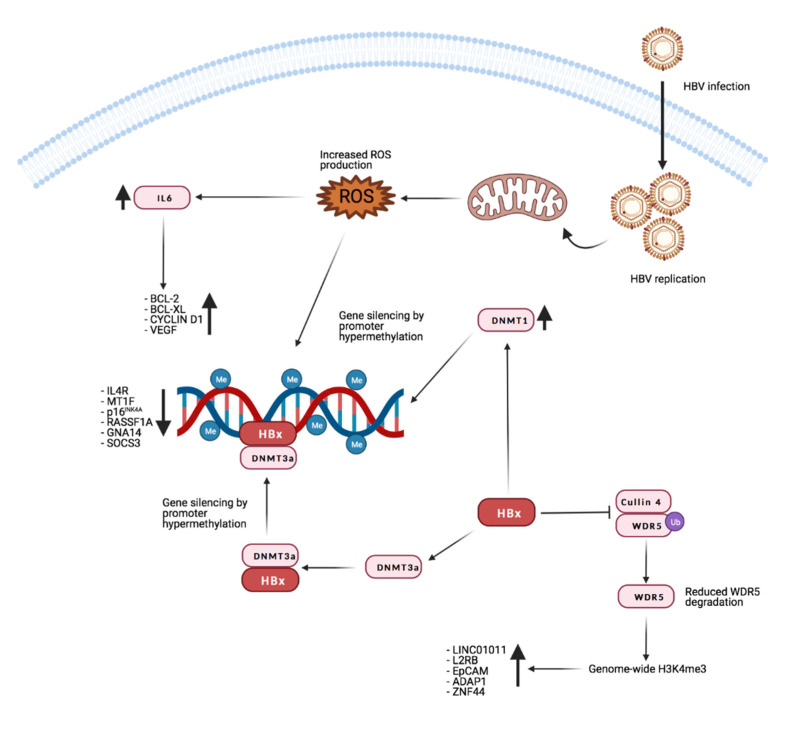
Effects of HBx on host methylation patterns. The viral protein HBx causes silencing of tumor suppressor genes by inducing repressive promoters hypermethylation through different mechanisms (see text for detailed description). At the same time, by inhibiting WDR5 degradation, it promotes the expression of target genes such as long intergenic non-protein-coding RNA 1011 (LINC01011), interleukin 2 receptor subunit beta (IL2RB), epithelial cell adhesion molecule (EpCAM), ArfGAP with dual PH domains 1 (ADAP1), and zinc finger protein 44 (ZNF44). Credits: Created with BioRender.com.

Recent research from Gao and colleagues shows that HBx promotes H3K4me3 by preventing the degradation of WD repeat domain 5 protein (WDR5), a core subunit of histone H3 lysine 4 methyltransferase complexes. Moreover, HBx can directly interact with WDR5 by binding with its α-helix domain, thus affecting WDR5 localization on the chromatin genome and promoting the expression of genes important for cancer progression [148]. Figure 2 reports the main known effects of HBx on gene expression through manipulation of the host’s genome methylation landscape.

By performing a genome-wide analysis Guerrieri et al. showed that HBx induces the activation of several cellular genes and miRNAs with a positive effect on processes like autophagy and endocytosis, while inhibiting the expression of targets that would be potentially detrimental to viral replication. This research was further expanded in a more recent paper showing that HBx interacts with the long non-coding RNA (lncRNA) DLEU2 to displace EZH2 from both the viral and host genome. Specifically, the authors identified six genes co-regulated by HBx, DLEU2 and EZH2 (TRIM13, CCNB2, DNMT1, PRC1, POLE2 and ZBTB34). The fact that they are all upregulated in HBV- related HCC tissues further highlights the importance of this mechanism [149].

Methylation is not the only histone modification altered by HBx. It has been shown that HBx can recruit the CREB/p300 complex to the promoter of genes that support HCC development. Upregulation of gene expression by HBx-induced histone acetylation was described for IL-8 and for proliferating cell nuclear antigen (PCNA) [150].

HBx can also induce degradation of host proteins through the Cullin 4A/DNA damage protein 1 (DDB1) E3 Ubiquitin Ligase Complex [151,152,153,154]. Specifically, HBx can target SMC5/6 to the aforementioned complex by acting as a bridge between DDB1 and SMC5/6, therefore inducing the ubiquitination of the latter. Interestingly SMC5/6 have been demonstrated to negatively impact transcription of viral cccDNA. For this reason the restoration of SMC5/6 function is being evaluated as a possible therapeutic approach to reduce the progression of liver carcinogenesis in HBV infected patients [155].

It has been shown that SMC5/6 colocalizes with nuclear domain 10 (ND10) in primary human hepatocytes, which suggests it acts as an intrinsic antiviral restriction factor that suppresses HBV transcription [156].

ND10 are spherical bodies present in the nucleoplasm linked to many functions like epigenetic regulation [157] and their dysregulation has been observed in HBV-infected cells [158]. Taking into account that the NSMCE2 subunit of the SMC5/6 complex has been shown to suppress cancer in mice [159], it is possible that the HBx mediated effects on ND10 and SMC5/6 could be important for HCC development.

Even though HBx is the main effector on the host genome, there is also evidence that the HBV core protein (HBc) can alter the expression of cellular genes by binding the cancer related promoter regions [160]. Intriguingly, a recent report also suggests that the episomal viral DNA may alter host chromatin organization. Moreau et al. showed that HBV DNA contacts preferentially CpG islands enriched for Cfp1, a factor required for HBV transcription [161]. Moreover, the CpG islands in contact with episomal viral DNA are associated with genes highly expressed and/or deregulated during HBV infection.

It is clear from these observations that HBV deeply alters cellular gene expression by using different epigenetic mechanisms.

## 6. Human Papillomavirus (HPV)

Human papillomavirus (HPV) is a non-enveloped tumor DNA virus with a genome of about 8 kb and is the agent of a common sexually transmitted infection globally. Although the infection clears, persistent infection leads to tumor development [162]. HPV infection occurs in the cutaneous or mucosal epithelium, especially in the genital tract.

Persistent infection accounts for 70% of cervical cancer and 90% of genital warts. Other neoplasms directly connected to HPV infection include anal, penile, vulvar, vaginal and oropharyngeal cancer [162].

Over 100 different strains have been identified of which HPV16 and 18 represent the most common high risk (HR) ones [163,164].

The characterization of the HPV genome has led to the identification of three distinct functional regions, specifically an early region which encodes for early proteins (E1–E7) necessary for replication and transcription of HPV DNA, a late region encoding for two late proteins (L1 and L2) that compose the capsid, and a long control region (LCR) which contains the early promoter and several transcriptional regulatory elements. The viral genome exists in the nucleus of infected host cell as an episome [165,166,167].

The E2 protein acts as a tether connecting HPV episomal DNA to the host genome through interaction with bromodomain-containing protein 4 (BRD4) [18,168]. In addition, both the E1 and E2 proteins are involved in ensuring the proper segregation of the viral genome during cell division [169].

The HPV genome can integrate into the host cell’s genome, and this is a critical part of the oncogenic process. Following the viral genome integration, loss of the E2 protein and often loss of E1, E4 and E5 [170], leads to constitutive expression of E6 and E7 [171].

In regard to their role in oncogenic transformation, E5, E6, and E7 are the most studied proteins [172], with the last two being the main contributors [173]. E5 has been shown to be required only in the early stages of tumorigenesis but is dispensable in progression and maintenance [174,175].

HPV E6 and E7 inactivate the tumor suppressors p53 and pRB [176], but their action extends to many aspects of tumor development, inducing cell proliferation, invasion, metastasis and angiogenesis, favoring genome instability and development of resistance to cell death [11]. Moreover, a growing number of reports indicate that they deregulate the epigenome of the host cells [177,178,179]. Both E6 and E7 modulate pathways that lead to degradation of the tumor suppressors p53 and pRb through physical interaction with different histone methyltransferases, acetyltransferases, and deacetylases, as summarized in Table 1.

Other important targets of these viral oncoproteins include the histone lysine demethylases 6A and 6B (KDM6A and KDM6B). E7 induces the upregulation of both demethylases and consequently increases the transcription of their downstream targets [185]. Specifically, the upregulation of KDM6A and KDM6B leads to decreased levels of the repressive trimethylation of lysine 27 of histone 3 (H3K27me3), which is necessary for the binding of Polycomb repressive complexes (PRCs) [185]. The mechanisms employed by E7 to upregulate these two demethylases are not clear. However, it is known that at least KDM6B upregulation is not dependent on HPV16 E7-mediated pRB degradation and consequent E2F activation.

An important downstream target of KDM6B is p16^INK4A^ which is found to be upregulated in cells expressing E7 [186]. Even though p16^INK4A^ is considered a tumor suppressor and isdownregulated in different cancers [187,188,189], its expression seems to be required in HPV induced tumors. Specifically, E7 can induce proteasome-mediated degradation of pRb by interacting with the cullin 2 ubiquitin ligase complex [190]. There is evidence suggesting that in the absence of pRb, cell survival requires overexpression of p16^INK4A^ because of its inhibitory effect on cyclin-dependent kinases 4 and 6 (CDK4/6) [186,191,192].

In addition, upregulation of KDM6A has been shown to induce the expression of homeobox (HOX) genes, thus deregulating a number of development and growth processes [185,193].

E7 can also upregulate enhancer of zeste homolog 2 (EZH2) a member of the Polycomb repressive complex 2 (PRC2), a methyltransferase responsible for mono-, di-, and tri-methylation of H3K27. While this may apparently sound counterintuitive in light of has been discussed so far, it is important to note that in these conditions AKT activity is increased. In fact, AKT can inactivate EZH2 by phosphorylation of serine 21 and therefore EZH2 overexpression does not increase PRC2 activity but rather favors the formation of the polycomb repressive complex 4 (PRC4) [194,195,196] which is involved in cancer and inflammation [197,198,199]. At the same time, E7 can bind and inactivate the B cell specific Moloney murine leukemia virus integration site 1 (BMI1), the main component of the polycomb repressive complex 1 (PRC1). Together with EZH2, BMI1 trimethylates and binds to H3K27 to maintain chromatin stability. This induces a global loss of H3K27me3 and consequent de-repression of target genes [195].

**Figure 3:**
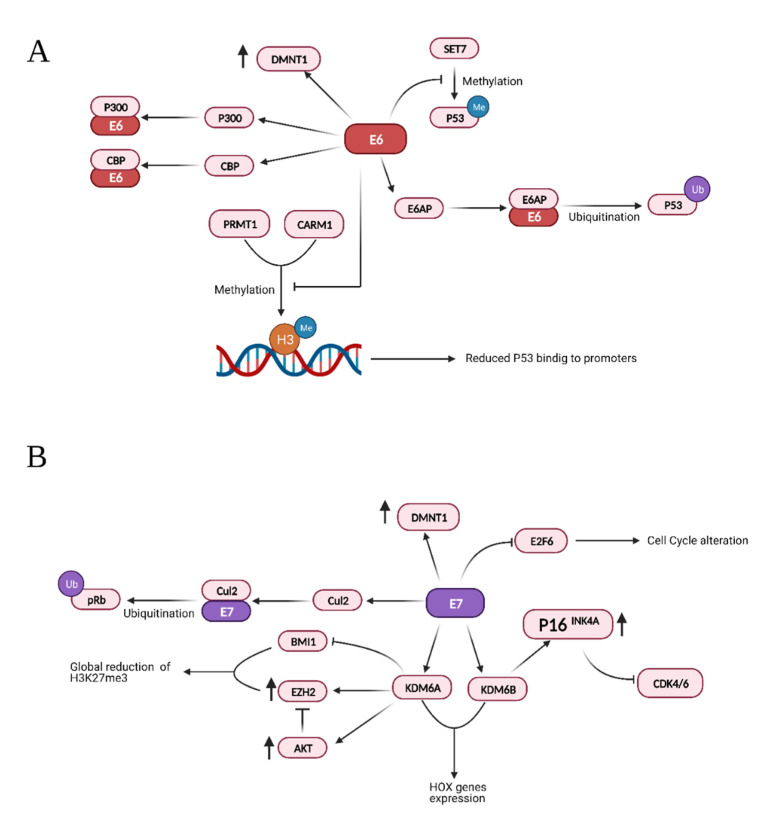
Summary of the effects and interactions of HPV protein E6 (**A**) and E7 (**B**)**.** Credits: Created with BioRender.com.

Additionally, HPV16 E7 can directly bind E2F1 and, as a result, enhances E2F1-mediated transcription [200]. It is known that E2F1 positively regulates the transcription of E2F6, which counteracts the transcriptional activity of E2F-responsive genes, negatively affecting cell cycle progression. However, HPV E7 also interacts with E2F6 through its C-terminal repression domain, thus relieving its transcriptional repression activity. This would keep the cells in an S-phase-competent state, which is required for the viral life cycle [201,202]. The main interactions and effects of the viral proteins E6 and E7 are summarized in Figure 3.

Finally, while the viral oncoprotein E6 and E7 are the most extensively studied, a recent report from Ren et al. investigated the oncogenic role of the episomal expression of the E2, E4 and E5 proteins [203]. Whole genomic expression analysis of pharyngeal and cervical cancers co-expressing E2, E4 and E5 showed a mechanism of carcinogenesis distinct from those found in tumors with HPV integration, and apparently characterized by fibroblast growth factor receptor (FGFR) pathway activation [203].

In conclusion, the analysis of the effect of HPV infection on the epigenetic landscape of the host cell reveals an intricate network of interactions between viral and host proteins.

## 7. Merkel Cell Polyomavirus (MCV)

Merkel cells, first identified by Friedrich Merkel in 1875, are located at the basal layer of skin epithelium cells and their development is driven by the atonal bHLH transcription factor 1 (ATOH1) [204,205].

Merkel cell carcinoma (MCC), also called neuroendocrine carcinoma of the skin, was first described in 1972 by Cyril Toker [206]. It is a rare aggressive tumor that manifests as a rapidly growing pink-red skin nodule, particularly but not exclusively on the face, head or neck. MCCs frequently metastasize to distant sites such as brain, lung, pancreas, lymph nodes, and bones [207,208]. Roughly 80% of all the MCC cases are positive for Merkel cell polyomavirus (MCPyV or MCV) and 20% of the cases are caused by UV-induced mutations [209].

MCPyV, a member of the *Polyomaviridae* family, contains a circular, double-stranded DNA genome [210,211] encoding structural proteins and the oncogenic large and small T antigens [210,211].

The MCPyV genome is often integrated into the MCC cells in a replication-deficient form (latency) due to truncation mutations in the viral large T-antigen [212,213]. It has been reported that, even in its truncated form, the MCPyV large T antigen can promote cell proliferation in both human and murine fibroblasts. In addition, the small T antigen showed oncogenic activity in transgenic mice [214,215].

Integration of MCPyV into the host genome is not part of the viral life cycle, as the virus does not contain integrase, and occurs through DNA repair mechanisms, such as non-homologous end joining (NHEJ) and microhomology-mediated end joining (MMEJ) [216].

The integration process is the main driver of tumorigenesis since it occurs near cancer-associated genes leading to the upregulation of oncogenes or the downregulation of tumor suppressor genes such as p53 and Rb [217,218,219,220]. This integration pattern clearly justifies the reason why the mutational burden is low in virus infected MCC tissues versus virus negative ones. However, no clear evidence has been documented for highly recurrent integration sites.

Systematic review of the published data regarding MCPyV integration sites revealed that chromosome 5 is the most recurrent locus for viral integration, detected in 21 out of 123 MCC cases [221]. The same study reported a unique case in which MCPyV integrated near the histone H3K4 methyltransferase KMT2D (MLL4).

A recent study, performed by Donglim Esther Park et al., reported that the small T antigen of MCPyV, in complex with MAX and EP400, binds to the promoter region of the Lysine-specific demethylase LSD1/KDM1A and components of the CoREST complex, such as RCOR2 [222]. In this regard, a panel of MCPyV positive MCC cell lines expressed higher levels of LSD1, RCOR2, and INSM1 compared to virus-negative cell lines and normal human foreskin fibroblasts (HFFs). ChIP-seq analysis showed enrichment of the viral small T-antigen at the promoter region of LSD1, INSM1, and RCOR2. Importantly, inhibition of LSD1 activity, either catalytically or by inhibition of its interaction with the CoREST complex resulted in reduced cell viability of virus positive cells. In addition, treatment of MCPyV positive cells with LSD1 inhibitors resulted in significant changes in gene expression patterns, while MCPyV negative cells showed a modest change in RNA levels after treatment. Intriguingly, inhibition of LSD1 resulted in an increase in the total H3K4me1 levels in MCV-positive cell lines. However, the authors did not report the global chromatin occupancy of H3K4me1 and H3K27ac, hallmarks of active enhancers, in response to LSD1 inhibition. Interestingly, a genome wide CRISPR screen identified KMT2C (MLL3), the only known H3K4 monomethyl-transferase, as one of the top genes enriched following LSD1 inhibition. Furthermore, treatment with LSD1 inhibitors of subcutaneous tumors in mice xenografted with MCPyV positive cells resulted in a significant reduction of growth and in alteration of protein levels.

Several studies have shown that lack of the Polycomb Repressor Complex 2 (PRC2) in the epidermis leads to the differentiation into Merkel cells due to upregulation of Merkel-specific differentiation genes [223,224,225].

Recent mutation analyses of MCC tissues revealed that virus-positive tumors have no mutational burden but show a loss in H3K27me3 levels [226]. The histone mark H3K27me3 is implemented by PRC2 and is associated with transcriptional repression and chromatin compaction [227,228]. Although the study did not provide a molecular explanation of how loss of H3K27me3 could correlate with poor prognosis, one might speculate that this histone mark might be lost at promoter regions of oncogenes, driving tumorigenesis without the need for driver mutations. On the opposite side, several studies have reported that in more than 50% of MCC tissues, enhancer of zeste homolog 2 (EZH2) is expressed at moderate/strong levels in primary tumors and is associated with poor prognosis [229].

These studies suggest that EZH2 functions as an oncogene by implementing H3K27me3 histone mark at tumor suppressor genes.

## 8. Kaposi Sarcoma-Associated Herpesvirus (KSHV)

Kaposi sarcoma-associated herpesvirus (KSHV), also known as human herpesvirus 8 (HHV-8), is a double-stranded DNA virus belonging to the family *Herpesviridae*, subfamily *Gammaherpesvirinae*. KSHV is associated with Kaposi sarcoma (KS), primary effusion lymphoma (PEL), and multicentric Castleman’s disease (MCD) [230]. Its oncogenic potential is well known and has been observed especially in immune-compromised subjects. Following infection, the virus can persist in the host in a latent or lytic state [231,232].

Several viral genes have been reported as presenting tumorigenic properties: latency-associated nuclear protein (LANA), LAMP, viral FLICE inhibitory protein (vFLIP), Kaposin, v-CyclinD, and viral interferon regulatory factors (vIRF) 1 to 7 [233,234,235]. These viral proteins affect different tumorigenic pathways by interacting with important oncogenes and tumor suppressors in mammals [236,237,238,239,240]. It has recently been demonstrated that v-CyclinD is critical for KSHV-infected human lymphatic endothelial cells to overcome replicative senescence, suggesting it may play an important role in KS tumorigenesis [241].

The viral protein LANA is critical for the persistence of the episomic viral genome during latency. LANA is responsible for both the replication of the viral episome during each cell division and for its correct segregation by acting as a tether to the host cell chromosome [242].

LANA binds to pRb, stabilizes and activates c-Myc, increases β-catenin-regulated gene expression (by inhibiting GSK3β) [243,244,245,246] and promotes cell survival through interaction with p53 [238,247,248].

One of the ways KSHV hijacks the epigenetic apparatus of the host cell is by the interaction of LANA with methyltransferases like DNMT3A, which are recruited to promoters, leading to transcriptional repression by hypermethylation [249]. Indeed, LANA was reported to induce DNA methylation at Sp-1 binding sites in the promoter of the TGF-β type II receptor, inhibiting TGF-β signaling [240]. Interestingly, TGF-β type II receptor levels are reduced in PEL, KS, and MCD. This observation, together with the fact that demethylating agents are reported to sensitize PEL lines to apoptosis, highlights the possibility that this epigenetic mechanism might be involved in the development and progression of KSHV-associated tumors [240]. So far, it has not been demonstrated that silencing of TGF-β type II receptor promoter is due to DNMT3A, as is the case for H-cadherin [249].

**Figure 4:**
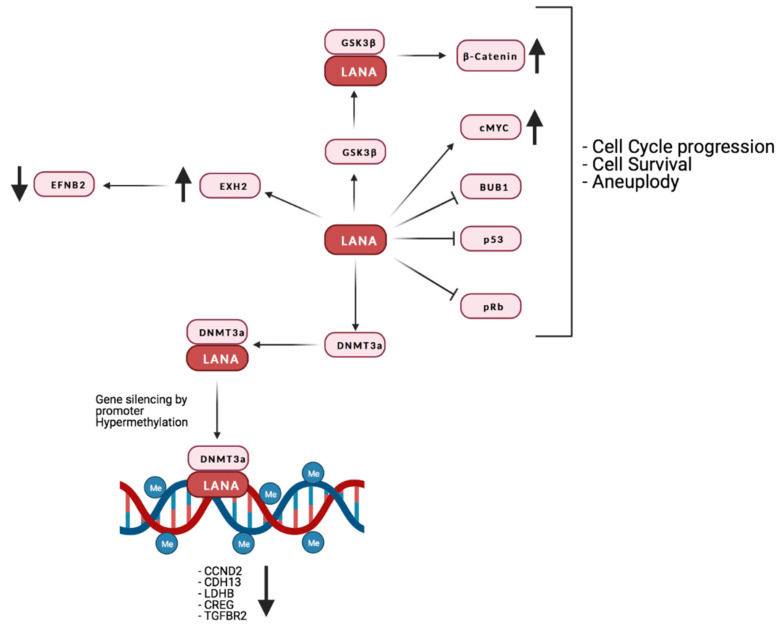
Summary of LANA’s interaction with the host’s proteins and their effect on the host’s gene expression. Credits: Created with BioRender.com.

Interestingly, a study from Shamay et al. suggested that KSHV might favor increased DNA methylation on a bigger scale than previously thought [249]. A number of studies investigated the interaction of LANA with host chromatin [250,251,252]. Even though the binding sites identified in these studies may partially differ, due to the use of a different cellular systems, they show that LANA binds to a far larger number of sites than previously thought. Moreover, binding is preferentially targeted to euchromatic loci, possibly due to LANA’s interactions with the H3K4 methyltransferase hSET1 or with BET [253,254,255,256]. It is also of interest that the expression of genes in the proximity of those binding sites does not seem to be affected, suggesting that methylation mediated silencing may be context specific. It is also possible that LANA binding may affect the expression of certain loci only in the presence of the activation of specific pathways. Lu et al. provided evidence that this could be the case for interferon gamma (IFNγ) regulated genes. In fact, these authors demonstrated not only a partial overlap between LANA and Stat1 binding sites within the promoters of IFNγ-regulated genes, but also an impaired response to IFNγ mediated activation [252]. It has also recently been suggested that KSHV infection induces alternative lengthening of telomeres (ALT), probably through LANA’s interaction with break-induced replication (BIR) factors [257]. Furthermore, together with vFlip, LANA can positively regulate the EZH2 transcription, thus upregulating the proangiogenic factor Ephrin-B2 [258]. A recent study also found that LANA may promote tumorigenesis by inducing chromosomal instability (CIN). Specifically, LANA inhibits the mitotic spindle checkpoint protein Bub1- mediated phosphorylation of histone 2A and cell division cycle protein 20 homolog (Cdc20), thus dislodging from the centromeres the Shugoshin-1 (Sgo1) and cohesin proteins, which are essential in chromosome cohesion during mitosis. Indeed, the displacement of Sgo1 impairs segregation of chromatids and leads to aneuploidy [259]. The main interaction of LANA with the host’s factors is reported in Figure 4.

Latency genes are not the only viral factors that can epigenetically affect viral and host chromatin. Among the lytic genes, it is important to mention the polyadenylated nuclear (PAN) RNA, a long non-coding RNA (lncRNA), expressed at low levels during latency but dramatically upregulated during the lytic phase [260]. PAN can interact with a number of epigenetic regulators, and can regulate the expression of both viral and cellular genes by interacting with the PRC2 components, EZH2, Suz12, and also with the H3K27-specific demethylases UTX and JMJD3, as well as with the H3K4-me3 methyltransferase MLL2 [260,261,262]. By tuning PRC repression, PAN can induce the expression of lytic viral genes and downregulate immune regulatory genes, acting in a context specific fashion.

Other lytic viral genes reported to alter host epigenetic regulators are vIL-6 and vIRF1 [263,264]. Specifically, vIL-6 binds gp130 and activates the JAK-STAT3 pathway, leading to DNMT1 upregulation and aberrant DNA methylation. On the other hand, DMNT1 expression is also increased by vIRF1 inhibition of p53 transcriptional activity, thus leading to increased expression of high mobility group box 2 (HMGB2) and cytidine/uridine monophosphate kinase 1 (CMPK1) genes, which are involved in cell motility and proliferation, and their upregulation is associated with poor prognosis in KS and other tumors.

vIRF1 has also been shown to block the formation of CBP/p300-IRF3 complexes by competing with cellular IRF3 for CBP/p300 binding. This inhibits IRF3-mediated transcription and signal transduction of type I interferon [265]. Moreover, vIRF1 can negatively affect TGF-β signaling by preventing the binding of Smad3/Smad4 complexes to DNA, thus suppressing IRF-1-induced CD95/CD95L mediated apoptosis [266,267].

On the other hand, vIRF3, which deregulates HDAC5 activity, seems to be essential for KSHV-Induced lymphangiogenesis, potentially opening new therapeutical opportunities [268].

Moreover, from a broader epigenetic perspective, vIRF3, together with cellular IRF4 and basic leucine zipper ATF-like TF (BATF), has been demonstrated to co-occupy super enhancers (SEs) of key survival genes in PEL cells. Functional experiments and transcriptome profiling following inhibition on these factors showed that this is critical for the survival and proliferation of KSHV-transformed B cells [269].

Recently, it has been demonstrated that vIRF4 regulates the host enhancer function during viral reactivation. In fact, during latency the host IRF4 effect on expression of enhancer RNAs (eRNAs) upregulates MYC. However, when shifting to the lytic phase, vIRF4 inhibits IRF4 and therefore downregulates MYC. This in turn facilitates lytic replication [270].

Interestingly, the virus-encoded bZIP family protein K8 (also known as K-bZIP) has recently been described to not only play an important role in viral DNA replication, but also to coordinate with non-coding RNA in order to act as a transcriptional repressor, and influence splicing and the host’s gene expression [271]. Since deregulation of non-coding RNAs plays an important role in oncogenesis, it has been suggested that the interaction of K8 with non-coding RNAs could contribute to neoplastic transformation [271,272].

Another recent study shed light on the relationship between KSHV oncogenic pathways and the extracellular growth environment. Naipauer et al., showed that KSHV-infected mesenchymal stem cells can form tumors when injected in nude mice, only when cultured in pro-angiogenic KS-like growth conditions. Compared to infected mesenchymal stem cells cultured under normal conditions, these cells show lower levels of repressive H3K27-me3 on viral genes, and changes of H3K27-me3 levels on different host promoters including VEGF, Toll-like receptor of INF signaling and p53. This suggests that the regulation of a number of pathways, together with the repression of innate immune response genes, is necessary to tolerate oncogenic KSHV lytic gene expression [273].

KSHV-induced oncogenesis involves a widespread manipulation of the host epigenome through a variety of mechanisms that are yet to be fully elucidated. The investigation of such mechanisms has been hindered by the lack of appropriate infection systems. In fact, most studies were performed using fully transformed cell lines following either de novo infection or ectopic expression of viral factors. Furthermore, some of the changes to the host chromatin are the result of interaction between constitutively expressed latency genes and transiently expressed lytic factors. Moreover, it is known that 90% of PELs also harbor EBV, which suggests that some of the observed epigenomic changes may be the result of the interaction of KSHV and EBV factors [274]. Interestingly a recent study from Wang et al., successfully employed H3K27ac HiChIP on both EBV-positive and EBV-negative PEL cell lines to generate PEL enhancer connectomes and link PEL enhancers to their direct targets [275], thus representing a first step in the identification of KSHV-specific effects on the host chromatin organization.

## 9. Human Cytomegalovirus (HCMV)

Human Cytomegalovirus (HCMV), also known as human herpesvirus type 5, is a member of the *Herpesviridae* family, sub- family *Betaherpesvirinae*. Its large genome is constituted by ≈235 kb of linear double stranded DNA and can encode for more than 165 open reading frames, four long non-coding RNAs, and several miRNAs [276].

HCMV can infect different cell types within its host, and the principal targets for its replication are fibroblasts, monocytes/macrophages, smooth muscle cells, epithelial cells, endothelial cells and neural stem cells [277,278,279].

While HCMV infection in adult immunocompetent subjects is mostly asymptomatic, its pathogenic role is well established in immunocompromised patients. Moreover, congenital HCMV infections are often cause of birth defects in newborns [280].

Currently, the role of HCMV in oncogenesis is still under investigation, however different reports show a strong association between HCMV and human cancers. Its oncomodulatory effect is explained by the production of viral proteins activating oncogenic pathways such as apoptosis inhibition, cell cycle progression and cell survival that favor malignant progression of tumors. Furthermore, HCMV can be directly implicated in neoplastic transformation as suggested by studies showing the ability of HCMV to induce genetic damage in infected fibroblast cultures [281,282,283], cellular transformation in prolonged cultures of HCMV-infected primary mammary cells (HMEC), and tumor formation in immunodeficient mice after xenotransplantation of the transformed HMEC cells [284].

HCMV particles, proteins and nucleic acids have been found in several cancers including breast, colon, prostate and ovary as well as in medulloblastoma, neuroblastoma, rhabdomyosarcoma and malignant glioma [285,286,287,288].

The pathogenic role of HCMV in glioblastoma (GBM) development is suggested by the significant association between poor prognosis and high levels of HCMV infection observed in GBM patients in terms of HCMV protein expression in tumor cells [289]. Furthermore, by in vivo experiments on a murine model of GBM, Krenzlin et al. reported that mice perinatally infected with murine CMV (MCMV) had significantly increased tumor growth and angiogenesis. The authors identified PDGF-D overexpression induced by CMV as an essential mechanism for pericyte recruitment, angiogenesis, and tumor growth. Interestingly, treatment with the antiviral drug cidofovir reversed the angiogenic phenotype and increased the survival of MCMV positive mice [290].

These data are further supported by the observation that in clinical trials the addition of the antiviral drug valganciclovir to standard therapy significantly prolonged survival in GBM patients [291,292,293].

Interestingly, the HCMV glycoprotein B (gB) is found expressed in primary GBMs and has been demonstrated to enhance invasiveness of glioma cells, as well as proliferation both in vitro and in vivo by inducing sustained phosphorylation of AKT, SRC, and PDGFRα [294]. Notably, anti-gB antibodies inhibited the invasiveness of patient-derived HCMV-positive glioblastoma cells, thus suggesting that targeting this protein could be of therapeutic relevance [294].

**Figure 5:**
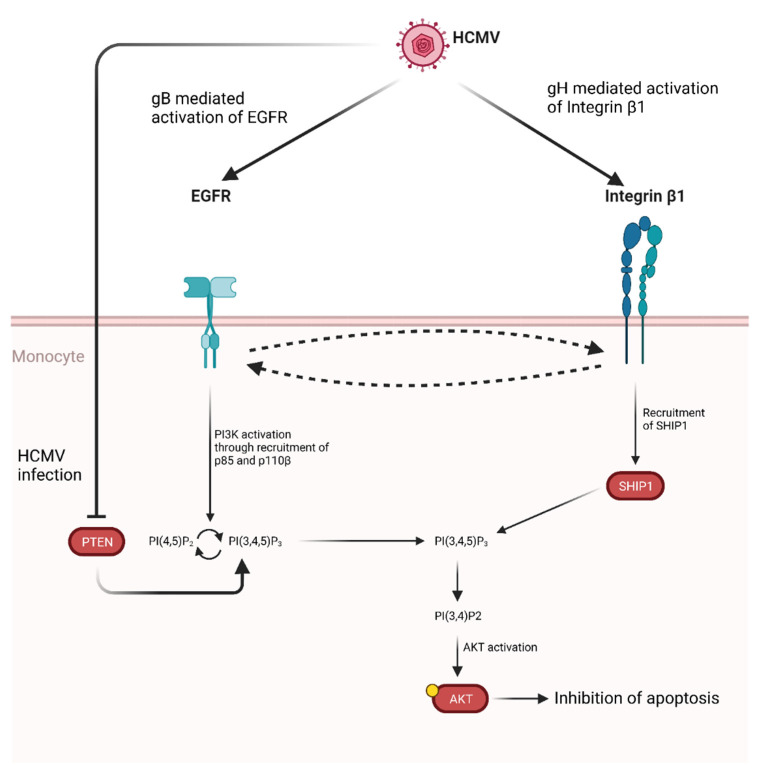
Proposed model for HCMV glycoprotein mediated AKT activation in monocytes. Following HCMV infection, gB and gH bind and activate EGFR and integrin β1, respectively. Cross-activation between receptors has been shown. Activation of EGFR through gB binding leads to PI3K activation through the recruitment of p85 and p110β. This, in turn, promotes the conversion of PI(4,5)P_2_ to PI(3,4,5)P_3_. This process is normally regulated by PTEN, which catalyzes the opposite reaction, however HCMV infection also inhibits PTEN, thus shifting the balance in favor of PI(3,4,5)P_3_ production [295]. At the same time gH binding to integrin β1 leads to SHIP1 recruitment which converts PI(3,4,5)P_3_ in PI(3,4)P_2_, thus activating AKT through a non-noncanonical pathway and ultimately up-regulating anti-apoptotic proteins. Credits: created with Biorender.com.

Activation of AKT induced by gB has also been demonstrated to promote monocytes survival [295,296]. Specifically, gB has been shown to act in tandem with HCMV glycoprotein H (gH) leading to atypical activation of Akt which ultimately leads to inhibition of apoptosis (Figure 5).

Furthermore, HCMV gene UL76 expression has been detected in human GBM cells and it is known to induce chromosomal breaks and induction of IL-8 through activation of the DNA damage response [281,297,298,299].

Similar to other oncoviruses, the oncogenic potential of HCMV differ between strains. Infection of primary human mammary epithelial cells (HMECs) with the HCMV-DB strain has been shown to result in a pro-oncogenic cellular environment characterized by decreased p53 activity, increased Rb phosphorylation, and enhanced telomerase activity. Moreover, cell proliferation was also increased due to c-Myc and Cyclin D1 upregulation as well as to increased AKT and STAT3 activation [284]. The same authors detected the viral lncRNA4.9 in infected HMECs cells, tumors isolated from xenografted NSG mice and biopsies of patients with breast cancer, thus suggesting that lncRNA4.9 could directly participate to the transformation process in this system. More recently, Nehme et al. screened different HCMV strains for their transforming potential and demonstrate that HCMV infection promotes polyploidy, stemness, and EMT/MET traits in HMECs [300].

Interestingly, HCMV proteins pUL123 and pUL122 have been detected in both breast cancer and GBM and are known to alter cell cycle progression by facilitating the entry into S phase [301,302,303]. Furthermore, the product of the viral gene UL111A (cmvIL-10) can bind to the IL-10 receptor and induce STAT3 activation and has been detected in different types of cancers [304,305,306,307,308,309,310]. The main viral product and their tumor-promoting activities are reported in Table 2.

Despite the evidence supporting an oncogenic potential of HCMV infection, some studies indicate a counteractive effect on tumor growth and progression. In fact, it has been reported that HCMV-infected MDA-MB-231 and SUM1315 breast cancer lines show a lower replication rate as well as migratory ability [338]. A similar antiproliferative effect is reported for HCMV-infected acute leukemic cells, inhibiting their proliferation and inducing apoptosis [339]. This result appears to be in line with the observation that patients affected by acute myeloid leukemia or non-Hodgkin lymphoma show a reduced relapse risk after early replicative HCMV infection following allogeneic stem cell transplantation [340,341,342]. Moreover, inhibition of in vivo growth and increase of cell apoptosis has been shown in xenograft experiments performed with HCMV-infected HepG2 cells [343].

The data discussed so far provide a complex picture of the role of HCMV infection in tumor development and maintenance. Although confounding factors such as difference in the oncogenic potential among viral strains as well as context specificity of the infection are present, there is strong evidence for the inclusion of HCMV in the group of human oncoviruses.

## 10. Therapeutic Approaches to Viral Tumors

**Figure 6:**
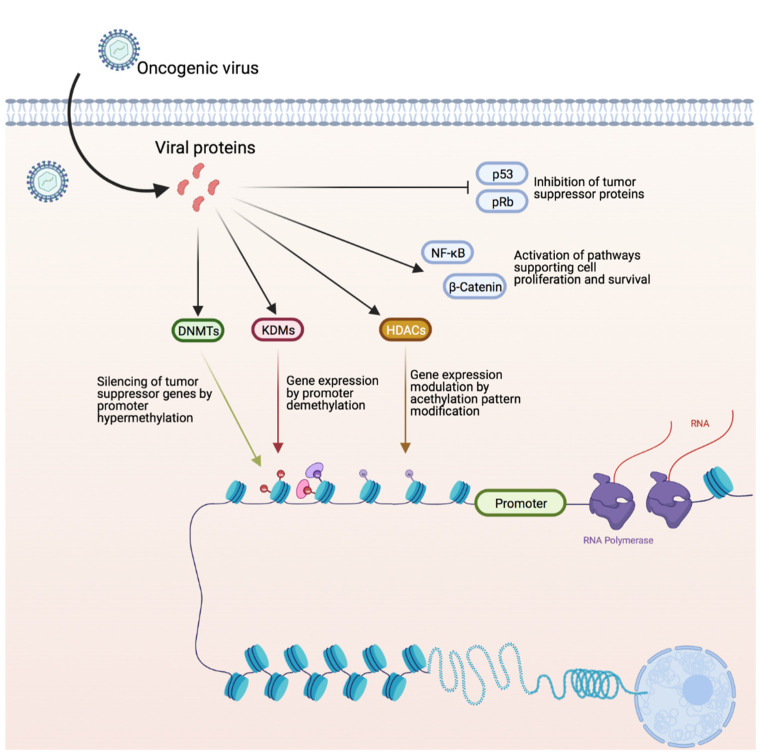
Common epigenetic alterations caused by oncogenic viruses. The host’s gene expression manipulated by interaction of the viral proteins with methyltransferases, demethylases and histone acetyl transferases. In general, these interactions result in silencing of tumor suppressor genes by hypermethylation of their promoters. At the same time, demethylases and acetyl transferases are targeted to specific loci to modulate their transcription. Viral proteins can also physically interact with the host tumor suppressor proteins inducing their degradation or preventing their interaction with target proteins. Similar mechanisms are employed to activate pathways to support proliferation or promote cell survival. Credits: Created with BioRender.com.

It is clear that alterations in the host’s epigenetic landscape play a pivotal role in sustaining viral replication and in driving oncogenic transformation. The main epigenetic alteration induced by oncogenic viruses are summarized in Figure 6.

Targeting epigenetic pathways in cancer is not a novel idea especially because of the reversible nature of epigenetic modifications [344]. So far, many compounds have been tested for their ability to inhibit epigenetic regulators.

### 10.1. Targeting DNA Methylation

As discussed before, oncogenic viruses deeply affect the host’s methylation patterns. A common feature is promoter hypermethylation, which results in silencing of tumor suppressor genes [345]. DNMTs inhibition is the most effective way to counteract abnormal DNA hypermethylation, thus potentially rescuing the expression of tumor suppressor genes and cell cycle regulators [346,347]. However, the hardest challenge in developing DNMTs inhibitors has been achieving good specificity, in order to avoid global genomic hypomethylation [348]. Among the DNMT inhibitors, 5-azacytidine (Azacitidine) and 5-aza-2′-deoxycytidine (Decitabine) have been beneficial for the treatment of acute myelocytic leukemia (AML) and myelodysplastic syndrome (MDS), both of which have been approved by the US FDA [349,350].

Both Azacitidine and Decitabine belong to the class of DNMT inhibitor defined nucleoside analogs. Briefly, their mechanism of action is to covalently bind to DNMT1 after incorporation into newly synthesized DNA [351]. Other methyltransferase inhibitors currently being evaluated are Zebularine and Guadecitabine, with similar mechanisms of action [349,352]. It was shown that decitabine inhibits tumor cell proliferation and up-regulates E-cadherin in EBV-associated gastric cancer [353], and there are case reports of successful treatments of EBV-positive large B-cell lymphoma with 5-azacytidine [354]. This provides a rationale for the use of DNMT inhibitors in EBV-associated cancers. Similarly, inhibition of DNMT enzymes shows promise in the treatments of HPV-positive cervical cancer. In fact, E6 and E7 expression was downregulated in HPV positive cancer cells following 5aza-2′-deoxycytidine treatment [355,356,357,358].

### 10.2. HDAC Inhibitors

Histone deacetylase inhibitors are also being developed and tested. Notably, HDACs not only acts on histones but also on other proteins involved in transcription or DNA repair [359]. The effect of HDACs inhibition has been studied in different types of cancer with promising results [360,361].

The precise mechanism of action for each inhibitor is not always completely understood, however the data obtained in clinical studies led to FDA approval for some.

So far, the FDA has approved Vorinostat, Romidepsin, Belinostat and Panobinostat. Panobinostat for combinatorial treatment of myeloma, the others for peripheral T-cell lymphomas. Tucidinostat, approved in China, is now in clinical trials [349,362,363].

Pracinostat (SB939), a pan-HDAC inhibitor, was investigated in combination with Azacytidine in AML patients, but the phase 3 study has recently been discontinued.

An interesting observation is that some cancers present mutations in HDACs resulting in resistance to inhibitors [364]. The study of such mutations could lead to the improvement of HDAC inhibitors efficacy.

Recently, it was shown that Vorinostat inhibits HPV-18 DNA amplification and induces apoptosis of HPV-infected raft cultures [307]. The same study reported similar results with Belinostat and Panobinostat.

In the context of MCC, HDAC inhibition not only promoted cell-cycle arrest and induced apoptosis, but it also restored HLA class-I surface expression both in vitro and in vivo, suggesting its potential use in combination with immunotherapy [143,365,366].

### 10.3. Targeting Histone Acetylation (Inhibitors for HATs and BETs)

DMNTs and HDACs are not the only epigenetic regulators that are studied as possible targets for cancer therapy. Histone acetyltransferases (HATs) are epigenetic enzymes that conjugate acetyl groups to lysine residues. On the other hand, bromodomain (BRD) containing proteins act as readers by recognizing acetyl-lysine and have important roles in gene expression regulation [367]. While many inhibitors targeting BRD-containing proteins have been studied in clinical trials, there are no clinical trials for HATs inhibitors. Competitive peptide inhibitors are available for PCAF, p300, and TIP60, however their use in biological systems is hampered by their size and limited membrane permeability [368,369]. For this reason, non-peptide small-molecule inhibitors are being developed, and some have shown good results in different types of cancer [370,371,372]. Among these, it is important to mention that C646, a selective p300 inhibitor, inhibits HPV E6-E7 transcription in cervical cancer. This is accompanied by p53 accumulation, suppression of cell proliferation, and apoptosis induction [373].

Other strategies focus on inhibiting the interaction of HATs with specific proteins rather than inhibiting their enzymatic activity. ICG-001 and PRI-724 are examples of such inhibitors and both compete with β-catenin for the interaction with CBP. Interestingly, it has been shown that the HTLV1 protein HBZ directly inhibits P300/CBP, leading to repression of p53-mediated transcription [374]. This seems to support the development of compounds able to specifically inhibit the interactions between viral proteins and HATs (or other epigenetic regulators). Bromodomain and extra-terminal motif (BET) protein inhibition is also being studied [375,376,377,378]. The mammalian BET family protein is comprised by BRD2, BRD3, BRD4 and BRDT. The first three are ubiquitously expressed, while the last is restricted to germ cells.

JQ1 and I-BET762 are the best known competitive inhibitors of BRD4. They bind to BRDs, thus blocking the recognition of acetylated lysine. Other compounds able to inhibit BRDs are MS417, OTX-015, RVX-208, OXFBD, I-BET151, PFI-1, MS436, and XD14 [379]. Recently, it was reported that the BRD4 inhibitor JQ1 induces depletion of E6 in HPV-induced cervical cancer cells, increasing the sensitivity to cisplatin even in cisplatin-resistant cell lines. More importantly, this effect was observed to be specific to HPV-positive cells, while cisplatin-induced death in HPV-negative cells did not increase [380], as observed in a previous study that used I-BET762 [381]. Similarly, BRD4 inhibition can suppress the tumorigenic effect of HTLV1 Tax protein by inhibiting Nf-kB signaling.

However, it is worth mentioning that JQ1 can induce HBV transcription, therefore there is a potential risk of HBV reactivation during therapy with BRD4 [382]. Dual inhibitors kinase/BET inhibitors and HDAC/BET are being evaluated as well as agents inducing the degradation of BET proteins like proteolysis-targeting chimeras (PROTACs) [383]. Recently, a report showed that concomitant inhibition of BRD4 and HDAC6 leads to synergistic anticancer effects in both HPV-positive and HPV-negative head and neck squamous cell carcinoma (HNSCC). The authors reported that the combination of ACY-241 and JQ1 induces a stronger response in terms of reduction of cell growth, induction of apoptosis and suppression of metastasis [384].

### 10.4. Inhibition of HMTs and HDMTs

The histone 3 lysine 79 (H3K79) methyltransferases DOT1L and EZH2, and the histone demethylase KDM1A (also known as LSD1) are also being studied as potential therapeutical targets [385].

The selective DOT1L inhibitor EPZ-5767 has been investigated in combination with cytarabine, daunorubicin and azacytidine in clinical trials for the treatment of ALL with MLL translocation [386,387].

Several inhibitors of EZH2 have been developed. 3-Deazaneplanocin A (DZNep) is one of the most studied and has shown anti-tumorigenic properties in oropharyngeal squamous cell carcinoma (OPSCC) cells in culture, with a stronger effect on HPV-positive cell lines [388]. The same authors confirmed this observation using other two EZH2 inhibitors: EPZ005687 and GSK-343. A more recent report also showed that EZH2 inhibitors may sensitize OPSCC cell lines to chemotherapy [389].

EPZ005687 and GSK-343 are also being investigated for the treatment of lymphoma and have shown to reduce H3K27 methylation in this context [390,391,392,393].

EZH2 inhibition is also being explored for ATL treatment. A recent article showed restoration of NDRG2 expression and reduction of cell proliferation in ATL cells following chemical or genetic inhibition of EZH2 [394]. Moreover, there is evidence in favor of EZH1 and EZH2 targeting in lymphomas [395].

Interestingly, the EZH2 inhibitor EPZ-6438 showed synergistic effects on growth inhibition when combined with HDAC inhibition in nasopharyngeal carcinoma (NPC) [396]. Moreover, EZH2 targeting seems to have a negative effect on the number of EBV LMP1-induced activated regulatory T cells, thus enhancing antitumor immunity in nasopharyngeal carcinoma [397]. Both DNMT1 and HDAC inhibitors showed increased efficacy when used in combination with LSD1 inhibitors [398] such as Tranylcypromine (TCP), ORY-1001, GSK2879552, and IMG-7289.

TCP showed promising effects against different types of leukemia by increasing H3K4me2 and the expression of genes associated with myeloid differentiation [399,400]. ORY-1001 can also promote the differentiation of leukemia cell lines, and has a better bio-availability than TCP [401].

Recently, LSD1 inhibition showed to be promising in the treatment of MCC. Specifically, LSD1 inhibitors negatively impact Merkel carcinoma cell growth in vitro and in vivo by promoting their differentiation toward normal Merkel cell fate [222,402,403]. Moreover, LSD1 inhibition sensitized hepatocellular carcinoma cells to sorafenib and regorafenib [404,405].

## 11. Conclusions

Oncogenic viruses establish a permanent latent infection sustained by the production of specific viral proteins, which interact with the cell environment, including the host epigenetic machinery to specifically deregulate pathways to their advantage such as cell metabolism, resistance to apoptosis, cell proliferation and innate immune response signaling. Epigenetic modifications largely alter host gene expression and can provide a common mechanism of virus-induced transformation.

In this review, we discussed the main epigenetic events involved in viral oncogenesis. In this context we also discussed the action mechanisms of the most relevant epigenetic drugs currently in use for the treatment of virus-induced tumors.

The best results using epigenome-targeted therapy have been obtained in hematological malignancies, while in solid tumors epigenetic drugs have shown to sensitize cancer cells to chemotherapy, immunotherapy or small molecule inhibitors. Even so, this therapeutic approach still lacks selectivity since it affects both normal and cancer cells.

Virus-induced cancers offer new opportunities for the development of small molecule inhibitors that could specifically block the interaction between viral and host factors, which would reverse virus-induced epigenetic modification with high specificity.

For this reason, characterization of the molecular events that lead to the epigenetic rewiring of host cells by oncogenic viruses is of pivotal importance.

## Figures and Tables

**Table 1:** Cellular factors that affect p53 and pRB pathways by interacting with HPV proteins E6 and E7.

Viral Protein	Cellular Interaction	Effect	References
	E6AP	P53 proteasome-mediated degradation	[180]
E6	CARM1 PRMT1	Prevents P53 binding to its target promoters	[179]
	SET7	P53 proteasome-mediated degradation	[179]
	p300/CBP	Increased p53 degradation	[181]
	p300/CBP	pRB degradation	[182]
E7	KAT2B	Inhibition of NF-kB Dependent activation of interleukin-8 (IL-8)	[183]
	CHD4	EnhancedHIF-1α- dependent transcription	[184]

**Table 2:** Human cytomegalovirus products and their reported oncogenic effect.

Viral Product	Cellular Target/Pathway	Effect	References
pUL16	NKG2D	Impaired NK cell recognition	[311]
pUL36	pro-caspase-8	Inhibition of apoptosis	[312]
pUL37x1	BAX	Inhibition of apoptosis	[19]
pUL76	S5a	Genomic instability	[313,314]
pUL82 (pp71)	- Rb - Daxx	- Increased cell proliferation - Increased incidence of mutations	[315,316]
pUL83 (pp65)	- NKp30 - - IFI16	Immune evasion	[317,318]
pUL97	- Rb - IFI16	- Increased cell proliferation - Immune evasion	[319,320,321]
pUL111A (cmvIL10)	- IL-10 receptor - STAT3	- Increased cell proliferation - Incresed cell migration - Telomerase expression - Immunosuppression	[308,322,323,324]
pUL122 (IE2)	- p53 - PI3K/AKT - Egr-1 - TGF-β	- Incresed cell proliferation - Cell cycle deregulation - Cell survival - Immunosuppression	[325,326,327]
pUL123 (IE1)	- p53 - Rb - IL-1 - Cyclin-E	- Increased cell proliferation - Cell cycle deregulation - Cell survival - Telomerase expression	[326,328,329,330]
pUS2	MHC-1	Immune evasion	[331]
pUS28	- HIF-1α - STAT3 - VEGF - NF-kB	- Cell proliferation - Cells survival - Tumor growth - Angiogenesis	[332,333]
miRs-UL112	MHC-1	Immune evasion	[334]
lncRNA4.9	PRC2	Increased cell proliferation	[284,335]
lncRNAβ2.7	GRIM-19	Resistance to mitochondria-induced cell death	[336,337]
